# Trauma surgery associations and societies: which organizations match your goals?

**DOI:** 10.1186/1752-2897-8-6

**Published:** 2014-06-14

**Authors:** Chad G Ball, Sean C Grondin, Colin Schieman, David V Feliciano, Elijah Dixon, Andrew W Kirkpatrick, Rao R Ivatury, Jeffrey P Salomone, Lawrence R Reed

**Affiliations:** 1 From the Departments of Surgery, University of Calgary, Foothills Medical Center, Calgary, Alberta, Canada; 2 McMaster University, Hamilton, Ontario, Canada; 3 Indiana University, Indianapolis, IN, USA; 4 University of Arizona, Phoenix, AZ, USA

## Abstract

This focused summary is a multi-institutional, multi-national, and multi-generational project designed to briefly summarize current academic trauma societies for both trainees and faculty alike. The co-authorship is composed of former and/or current presidents from most major trauma organizations. It has particular relevance to trainees and/or recent graduates attempting to navigate the multitude of available trauma organizations.

## 

Vigorous participation and active involvement in surgical subspecialty organizations is essential to both the advancement of the field itself, as well as to the personal development of individual surgeons and trainees. These societies provide members and presenters with the opportunity to meet and reconnect with mentors, absorb the latest surgical science and techniques, exchange ideas, travel worldwide, and perhaps most importantly, present their work in a peer-reviewed arena ripe with comments, critique, and feedback. This avenue for advancement and networking also extends beyond the actual organizational meetings and conferences. More specifically, surgical subspecialty organizations provide opportunities in the realms of quality improvement (databases, publication of guidelines, political lobbying), research (i.e. registries, multicenter trials, and grant funding), administration (committees), and education (curriculum design and revision, shared educational resources, collaborative skills laboratories, internet-based rounds and continuing medical education events).

In addition to the benefits listed above, many of these organizations shape the future and direction of their subspecialties as well. This applies to the clinical, administrative, and educational aspects of our craft and is especially evident within the field of trauma and acute care surgery. Caring for these challenging patients represents the core of general surgery and is therefore, one of the few philosophies that binds all of us together in a time of increasing fragmentation. Given that injury is a global public health epidemic, improving the care of trauma patients, both locally and internationally, is a fundamental goal of each of these organizations. Perhaps most importantly, these societies also posses tremendous value in enhancing an individual surgeon’s maturation on numerous levels. These include, but are not limited to, strengthening political, administrative and leadership skills beyond the immediacy of one’s own institution. They also play a strong role in maintaining competence and continuing medical education relevant to all surgeons whether novice or expert.

The goal of this review is to provide both surgeons and trainees with a brief overview of many currently operating trauma and acute care surgical organizations (Table 1).

**Table 1:** Trauma society characteristics

**Organization**	**Dominant goals and potential surgeon interests**
AAST	Trauma leadership within the United States & globally
	Scientific trials & continuing education
EAST	Aimed at the career development of young surgeons
	Academic development, leadership training & science
WTA	Balance between clinical trauma care & winter activities
	Strong scientific content with family fun environment
TAC	National multidisciplinary trauma care & collaboration
	Inclusive of prehospital, nursing & registry colleagues
PTS	Improving clinical trauma care in the Americas
	Continuing education and liaising for all surgeons
IATSIC	Multi-continental link amongst trauma care providers
	Medical education, science & leadership opportunities
ESTES	Large, widely inclusive, trauma care organization Encompasses most national European trauma associations

## Trauma surgery organizations based in North America

### American association for the surgery of trauma (AAST)

The AAST was founded in 1938 as the American Association for Traumatic Surgery with its first annual meeting on May 8, 1939 [1]. Today the membership approximates 1200 surgeons. The Association’s primary goals are advancement of research and education through publications in its highly cited journal (The Journal of Trauma and Acute Care Surgery - JOT). Other activities of the AAST include awarding of research grants, directing multicenter trials, education on-demand summits and retreats, and monthly nationwide educational webcast teleconferences. This organization has provided a consistent forum for trauma leadership from the United States to help shape the scope and nature of the trauma, acute care surgery, and critical care subspecialty over the past decade. This organization remains the heart of progress and vision on a global scale.

The annual meeting of the AAST has over 1000 attendees who meet in various locations across the United States in the month of September. This conference is intermittently held as a joint meeting with other international trauma societies. The meeting includes scientific content via concurrent sessions, invited lectures, topic focused breakfast or luncheon encounters, and poster sessions. Presentations at the AAST are submitted to the JOT for publication.

Membership in the AAST is restricted to sponsored surgeons and selected research scientists who have made significant contributions to the field of trauma surgery in either clinical, research, administrative, and/or educational work. The AAST is the flagship trauma organization in the United States and Canada, and remains one of the most respected in the world.

### Eastern association for the surgery of trauma (EAST)

The primary mission of EAST is to provide a forum for academic development and leadership training for young surgeons interested in trauma, surgical critical care, and acute care surgery [2]. In addition to scholarship and fellowship, EAST actively promotes trauma prevention, research and improvements in trauma system design. EAST has a large membership (1600), most of whom attend the annual conference in January (often in a family friendly resort in Florida). The meeting is an easy blend of science and camaraderie in an informal setting. Half the membership is considered active (board-certified surgeons). These members graduate to senior status at age 50, resulting in a continued opportunity for surgeons to assume leadership roles at an earlier age. Surgical residents and fellows may join as Provisional members. Additionally, 10% of EAST’s membership (Associate members) is non-surgeon physicians and other allied health personnel (nurses, mid-level providers and paramedics). Conference proceedings (including injury prevention) are published in the JOT.

EAST is also renowned for producing detailed and well-received clinical practice guidelines (CPGs). While the CPGs have traditionally spanned all areas of clinical traumatology, more recent additions have focused on topics in surgical critical care and emergency general surgery. EAST is clearly an organization dedicated to launching the academic careers of young surgeons and investigators in a fun and family-friendly environment.

### Western trauma association (WTA)

The first meeting of the WTA was held in 1971 at Vail, Colorado [3]. The driving goal for the 125 members from multiple specialties remains improving the care of injured patients through an annual 5-day diverse scientific program. This core value is synergistic with the spirit of collegiality and family in an outdoor recreation setting. The WTA annual conference is held at a ski resort west of the Mississippi river in February or March. These meetings are legendary for mixing science, recreation and friendships among both junior and senior surgeons alike. Podium presentations are submitted to the JOT.

The WTA has long been known for presenting and publishing evidence-based multicenter trials. In recent years, algorithms describing clinical care have been developed and published as well. The WTA is an organization with a unique combination of high-level science, outdoor recreation, family activities, and kinship in a spectacular venue.

### Trauma association of Canada (TAC)

The TAC was formed in 1983 at a meeting of the Royal College of Physicians and Surgeons of Canada as a maturation of the ad-hoc Trauma Committee of the Canadian Association of General Surgeons (CAGS) [4]. Although it stands independently, TAC has met jointly with the AAST, and continues to meet with the Australasian trauma society on a 4-year cycle. From the beginning, TAC’s dominant goal has been the inclusion of all specialties and disciplines that care for the injured patient. This initially included members from the Canadian societies for orthopedics, neurosurgery, emergency medicine, plastic surgery, urology, pediatric surgery, critical care medicine, and anesthesiology. TAC has since expanded to include the Interdisciplinary Trauma Network of Canada, which encompasses nurse-practitioners, clinical nurse specialists, trauma educators/clinicians, paramedics, clinical trauma nurses, researchers, data registry specialists and military personnel and medics. This policy has grown its membership to 270 people. Given the extensive multidisciplinary nature of TAC membership, scientific sessions at the annual meeting are broad and interesting. The TAC conference is typically a ski-based meeting that rotates amongst Whistler, Banff, and eastern Canada. In addition to advancing the national trauma system, TAC is also associated with Accreditation Canada with the challenging mandate to ensure evidence-based care in a large national geographic distribution. Manuscripts are submitted to the JOT (TAC is an affiliated society) or the Canadian Journal of Surgery.

## Large global trauma and acute surgery organizations

### Panamerican trauma society (PTS)

The PTS was founded in Bogota in 1986 with an initial goal of improving trauma care in Latin America [5]. The society has grown tremendously in the past 25 years with inclusion of emergency medicine, intensivists, nurses, and paramedics from across all of the Americas. The membership approximates 1000 surgeons and the leadership includes senior surgeons from North, Central and South America. The PTS also acts as the parent organization to several trauma focused organizations in Mexico, Ecuador, Columbia, Panama, Bolivia, Peru, and Argentina.

The PTS produces 3 to 4 issues of a newly constituted journal of clinical importance (Panamerican Journal of Trauma, Critical Care and Emergency General Surgery) with a multinational editorial board and excellent scientific content. Dominant initiatives of the PTS include: (1) developing comprehensive trauma care guidelines, (2) showcasing the vast trauma experience of South American surgeons, (3) developing international programs in injury treatment and prevention, (4) creating new paradigms applicable to native South American countries and (5) stimulating young surgeons to focus their attention on trauma care.

The annual PTS conference is typically held in November at a favorable location in Central or South America. It includes both scientific and expert opinion based sessions.

### International association for trauma surgery and intensive care (IATSIC)

Since its origin in 1989, IATSIC has functioned under the umbrella of the International Society of Surgery (ISS) [6]. Like TAC and PTS, it is multidisciplinary in nature and includes trauma and acute care surgeons, emergency medicine physicians and intensivists. Its membership is very broad and entirely multinational. It was founded as the first true forum for international exchange and creation of trauma care standards on a global basis. The primary goal of IATSIC remains communication and education amongst a community broadly defined as traumatology.

The biannual IATSIC meeting combines scientific sessions with lively debates and educational sessions in exotic international destinations. In addition, IATSIC is particularly focused on inclusion of resource challenged countries and trauma systems. It is well known for its Definitive Surgical Trauma Care (DSTC) course, one of the best of the genre. All manuscripts are submitted to the World Journal of Surgery. IATSIC is a mature, international organization with a focus on trauma care adapted to a variety of health care systems around the world.

### European society for trauma and emergency surgery (ESTES)

Since its origin in 2007 following the union of EATES (European Association for Trauma & Emergency Surgery) and ETS (European Trauma Society), the stated goal of ESTES has been to enhance the quality of care for acutely ill patients with surgical needs, through scientific discussions, research and disseminating recent clinical knowledge [7]. This broad society also acts as the umbrella organization to 28 European national injury societies. Membership has now reached well over 10,000 surgeons who specialize in the care of injured patients, making it the largest organization of relevance. The yearly scientific meeting (European Congress for Trauma and Emergency Surgery) is held in interesting countries across Europe with a dominant focus on knowledge dissemination. This goal is further enhanced by both postgraduate courses and international fellowships. Furthermore, ESTES also offers a European Trauma Examination which is content specific and remains unique across the globe. The ESTES also publishes a bimonthly peer-reviewed journal with a focus on clinical algorithms and scientific research for both trauma and emergency surgery. ESTES is strongly affiliated with multiple European, Global and American (AAST) counterparts, making it the preeminent society for the care of injured patients within Europe.

### German society society for trauma surgery (Gsts)

This extremely large (over 4,500 members) national trauma care organization within Germany continues to strive to ensure that every severely injured patient receives gold standard care in accordance with high quality standards [8]. This association has an annual meeting within Germany that is attended by over 11,000 delegates and represents one of the largest injury-related congresses in the world. GSTS is structured similarly to other national trauma societies with the aim of improving the quality of injury care via enhanced communication, creation of care standards, and improved cooperation on an organized national platform. This has included, but is not limited to, creating regional care directives, regional trauma referral centers, standardization of emergency resuscitation environments, offering medical education programs (DSTC, ATLS), and creating a national trauma registry. This organization continues to be a model for national societies throughout the world.

## Other organizations that should be considered for membership

*Society for Critical Care Medicine* (*SCCM*) [9].

*Canadian Critical Care Society* (*CCCS*) [10].

*European Society of Intensive Care Medicine* (*ESICM*) [11].

*American Trauma Society* (*ATS*) [12].

*Australasian Trauma Society*[13].

*South African Trauma Society* (*SATS*) [14].

*World Congress of the Abdominal Compartment Syndrome* (*WCACS*) [15].

*Japanese Association for The Surgery of Trauma* (*JAST*) [16].

*Turkish Society for Trauma and Emergency Surgery*[17].

### Which trauma organization should you join?

While this discussion of trauma organizations is not complete, it does identify most mature and large societies for clinicians and trainees focused on the surgical care of injured patients. Given that each society possesses a slightly different flavor and set of core values, surgeons and trainees should target the society that best aligns with their goals and interests. As a result, many surgeons belong to multiple organizations. These might include large national societies such as the American, German or South African trauma societies to assist the delegate in improving deliverable care within their own country on a coordinated nationwide front. Some surgeons might also join associations to generate a more broad international perspective on both clinical care and trauma system processes (e.g. ESTES, IATSIC, AAST, PTS). Alternatively, some clinicians may select a society for a particular mix of social, activity and meeting location details (WTA, TAC, IATSIC). From a junior perspective, the scientific quality, career development and international consensus building of the AAST, EAST and IATSIC respectively might be of particular relevance. Ultimately, selection is a very personal decision that may include a combination of national pride, scientific prowess, continuing education and/or social interaction. Membership, as well as personal time allotted to serving the organization itself, should be re-evaluated on a regular basis as the surgeon and the society evolve over time. Some members will aspire for occasional casual involvement and utilization of web-based materials while others will become involved to the highest levels of executive function. In summary, the trauma community is fortunate to have a large constellation of well-developed and focused international trauma organizations that will match any individual clinician’s goals.

### Consent

No patient consent was required in the construction of this review given the paucity of information relevant to any patients.

## Competing interests

The authors declare that they have no competing interests.

## Authors’ contributions

CGB and CS were responsible for drafting, editing and completing the manuscript. SCG, DVF, ED, AWK, RRI, JPS, and LRR were each responsible for drafting the manuscript. All authors read and approved the final manuscript.

## References

[B1] American Association for the Surgery of Trauma 2013 http://www.aast.org/Default.aspx, February 20, 2013

[B2] Eastern Association for the Surgery of Trauma 2013 http://www.east.org/, February 20, 2013

[B3] Western Trauma Association 2013 http://westerntrauma.org/, February 20, 2013

[B4] Trauma Association of Canada 2013 http://www.traumacanada.org/, February 20, 2013

[B5] Panamerican Trauma Society 2013 http://www.panamtrauma.org/, February 20, 2013

[B6] International Association for Trauma Surgery and Intensive Care 2013 http://www.iatsic.org/, February 20, 2013

[B7] European Society for Trauma and Emergency Surgery 2014 http://www.estesonline.org/news/26/welcome, May 14, 2014

[B8] German Society for Trauma Surgery 2014 http://www.dgu-traumanetzwerk.de, May 14, 2014

[B9] Society for Critical Care Medicine 2013 http://www.sccm.org/Pages/default.aspx, February 20, 2013

[B10] Canadian Critical Care Society 2013 http://www.canadiancriticalcare.org/, February 20, 2013

[B11] European Society of Intensive Care Medicine 2014 http://www.esicm.org, May 14, 2014

[B12] American Trauma Society 2013 http://www.amtrauma.org/ February 20, 2013

[B13] Australasian Trauma Society 2013 http://www.traumasociety.com.au/, February 20, 2013

[B14] Trauma Society of South Africa 2013 http://www.traumasa.co.za/, February 20, 2013

[B15] World Society of Abdominal Compartment Syndrome 2013 http://www.wsacs.org/, February 20, 2013

[B16] Japanese Association for the Surgery of trauma 2014 http://www.jast-hp.org/en/top/greeting.html, May 14, 2014

[B17] Turkish Society for Trauma and Emergency Surgery 2014 http://www.travma.org.tr/eng/page.aspx?menu=544, May 14, 2014

